# Supplementary material for: A NIR-Fluorochrome for Live Cell Dual Emission and Lifetime Tracking from the First Plasma Membrane Interaction to Subcellular and Extracellular Locales
Source: Molecules. 2024 May 24;29(11):2474. doi: 10.3390/molecules29112474 (PMC11174088; doi:10.3390/molecules29112474)
Supplement: Supplementary file 1 [file molecules-29-02474-s001.zip › DOShea SI_Revised.pdf]

Supplemental Information

A NIR-Fluorochrome for Live Cell Dual Emission and Lifetime Tracking from the First Plasma Membrane Interaction to Subcellular and Extracellular Locales

Eden Booth[1], Massimiliano Garre[1], Dan Wu[1], Harrison C. Daly[1], Donal F. O'Shea[1]*

[1] Department of Chemistry, Royal College of Surgeons in Ireland (RCSI), Dublin 2, Ireland

Contents

**Figure S1**	2
**Figure S2**	2
**Figure S3**	2
**Figure S4**	3
**Figure S5**	3
**Figure S6**	4
**Figure S7**	5
**Figure S8**	6
**Figure S9**	6
**Figure S10**	7
**Figure S11**	8
**Movie legends**	9

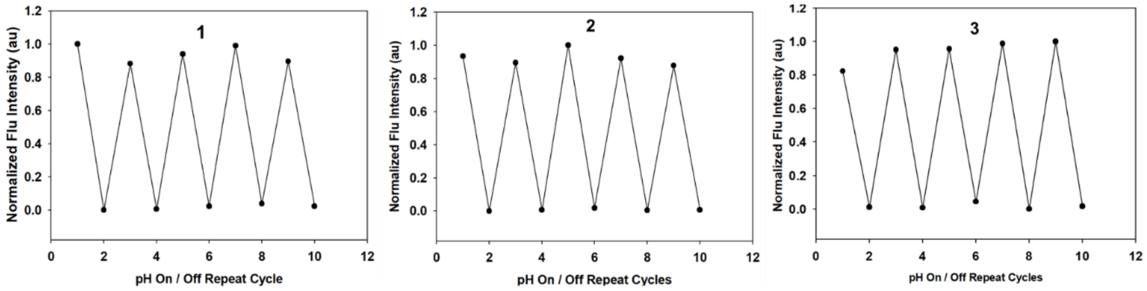

SI Figure S1. Fluorescence response at 720 nm to a repeating cycle of acidification (pH 2) and neutralization (pH 7) for **1**, **2**, and **3**.

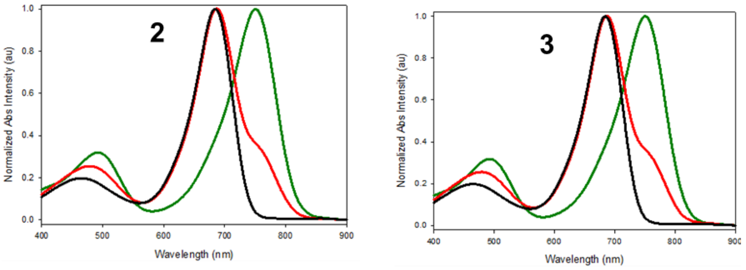

Figure S2. Normalized absorbance spectra at pH 7 (green trace), pH 4 (red trace), pH 2 (black trace) of **2** and **3**.

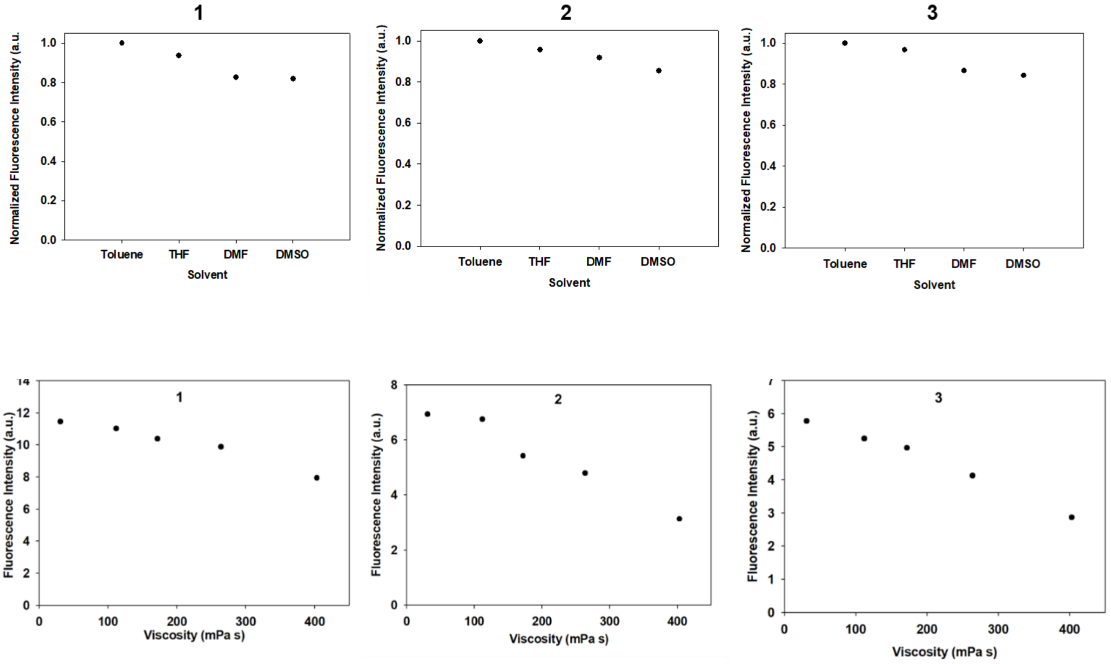

Figure S3. Polarity and viscosity fluorescence properties of **1**, **2** and **3.** Top: Plot showing fluorescence intensity of each fluorophore in toluene, THF, DMF and DMSO. Bottom: Plot showing fluorescence intensity of each fluorophore in different solvent viscosities.

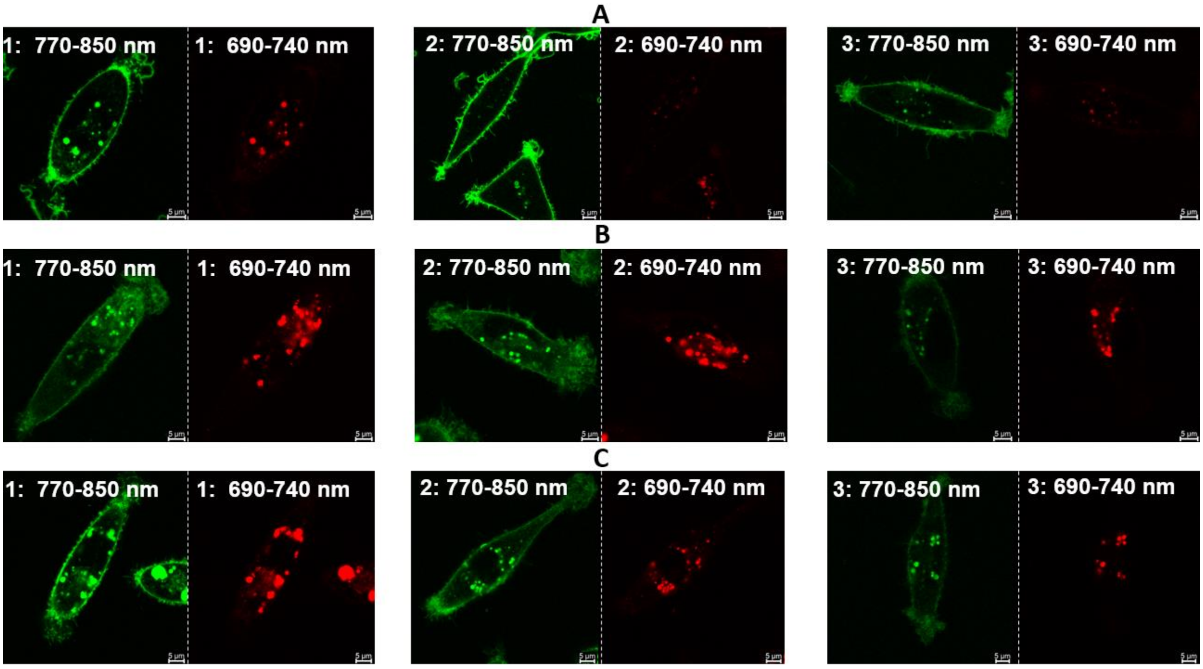

Figure S4: Time course of dual colour imaging of live MDA MB 231 cells following treatment with 2 μM concentration of **1** (left), **2** (middle) and **3** (right) at (A) 30 min. (B) 2 h and (C) 5 h. Images acquired using excitation at 760 nm with collection from 770-850 nm (green) or excitation at 680 nm and collection between 690 -740 nm. Scale bar 5 μM.

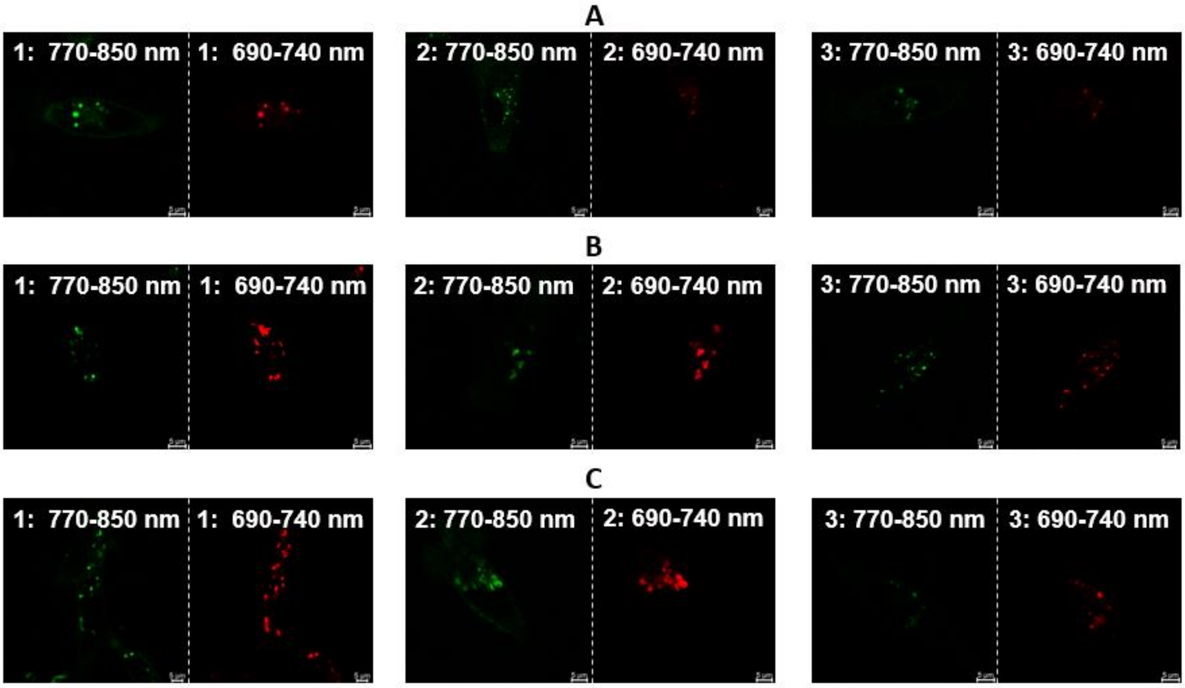

Figure S5. Time course of dual colour imaging of live MDA MB 231 cells following pulse-chase treatment with 2 μM concentration of **1**, **2** or **3** at (A) 30 min. (B) 2 h, and (C) 5 h. Images acquired using excitation at 760 nm with collection from 770-850 nm (green) or excitation at 680 nm and collection between 690 -740 nm.

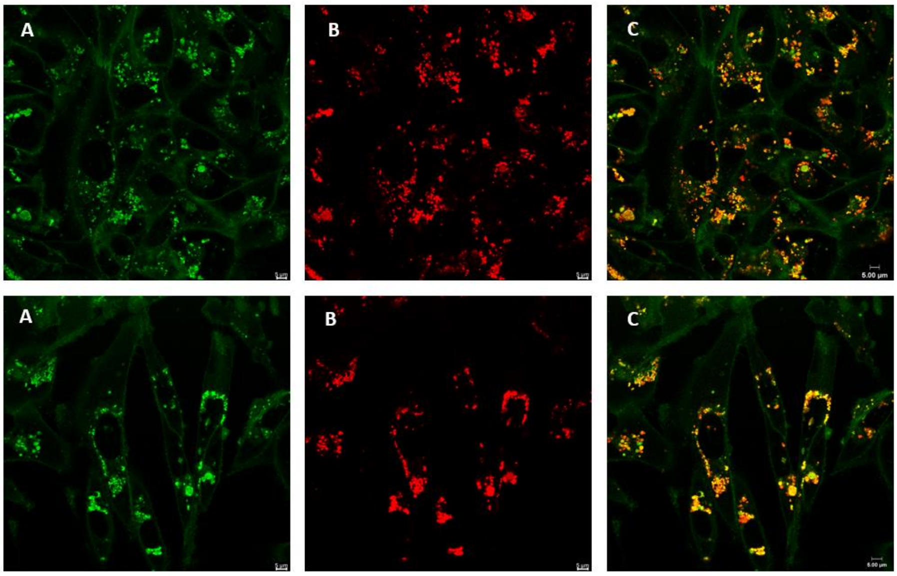

Figure S6. Representative confocal images of MBA MD 231 cells containing **1** following 48 h efflux. (A) Cells imaged using 760 nm excitation and 770-850 nm detector collection. (B) Cells imaged using 680 nm excitation and 690-740 nm detector collection. (C) Overlay of images A and B. Scale bar 5 μM.

Fluorophore 1

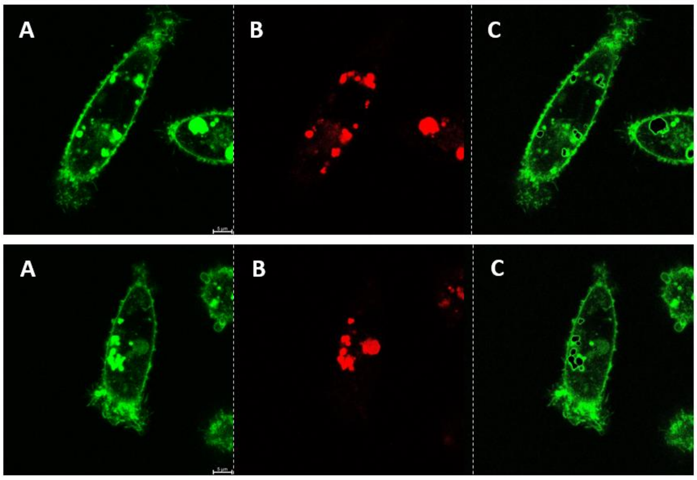

Fluorophore 2

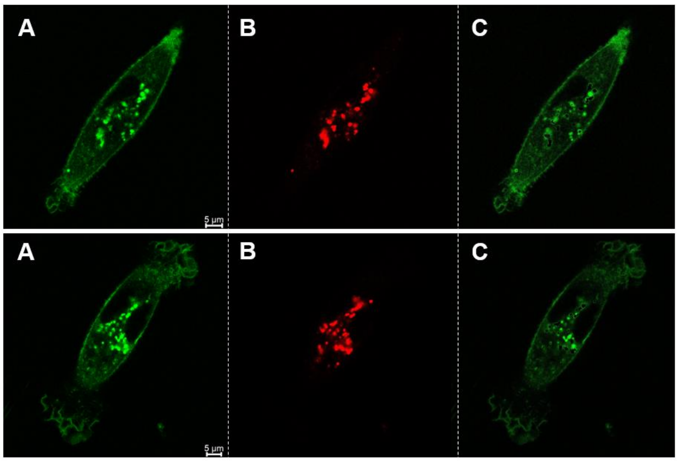

Fluorophore 3

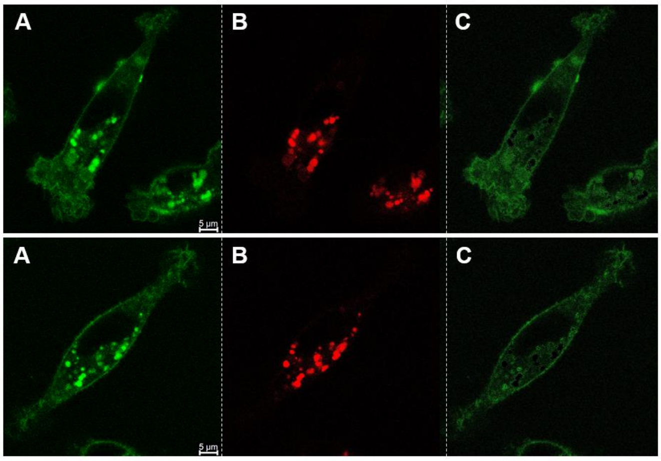

Figure S7. Visualization of intracellular non-acidic vesicles via image subtraction in live MDA MB 231 cells following treatment with 2 μM **1** (top panel), **2** (middle panel) or **3** (bottom panel) for 2 h. (A) Image acquired using excitation at 760 nm with collection from 770-850 nm (green). (B) Image acquired using excitation at 680 nm and collection between 690 -740 nm. (C) Resulting imaging following the subtraction of image (B) from (A). Scale bar 5 μM.

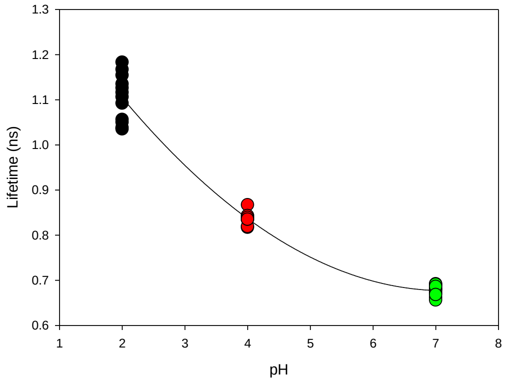

Figure S8. Phasor lifetimes of **3** in complete DMEM at pH 2 (black), pH 4 (red), and pH 7 (green) (n = 10).

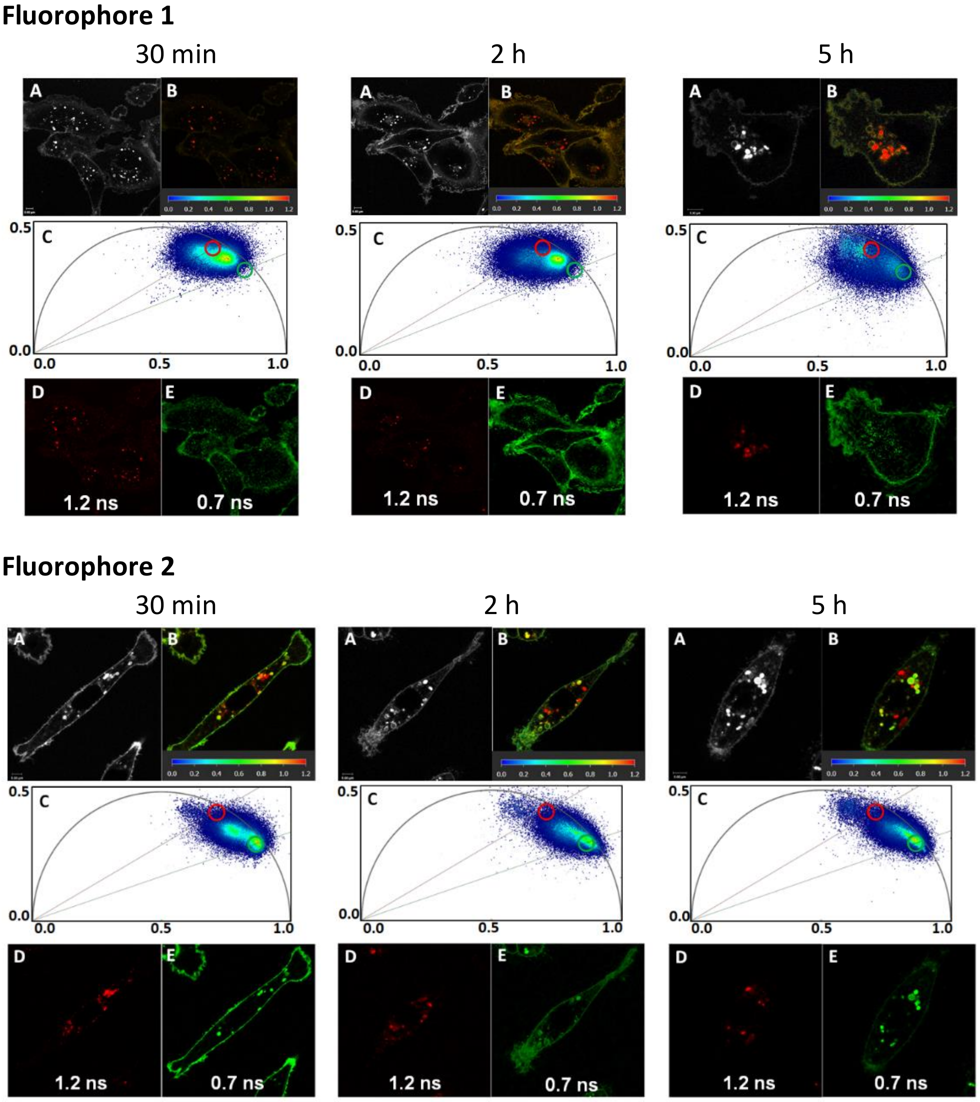

Figure S9. FLIM imaging with phasor analysis of live MDA MB 231 cells following treatment with 2 µM of **1** (top panel) and **2** (bottom panel) at 30 min, 2 h and 5 h. (A) Intensity images at specific time point (B) FLIM image with lifetime heat map at specific time points. (C) Phasor plots at specific time points with phasor lifetimes of 1.2 ns (red circle) and 0.7 ns (green circle) shown. (D and E). Scale bar 5 µM.

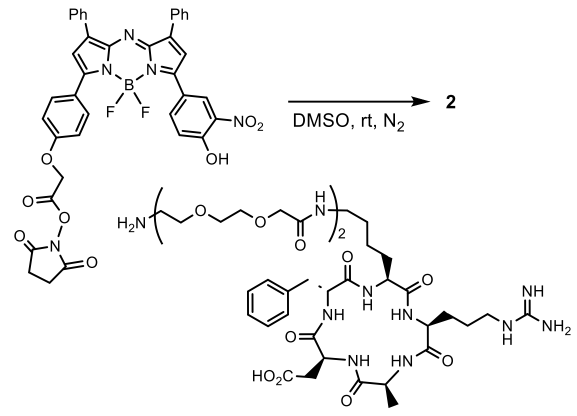

Figure S10. Synthetic scheme to fluorophore **2**

^{1}H NMR

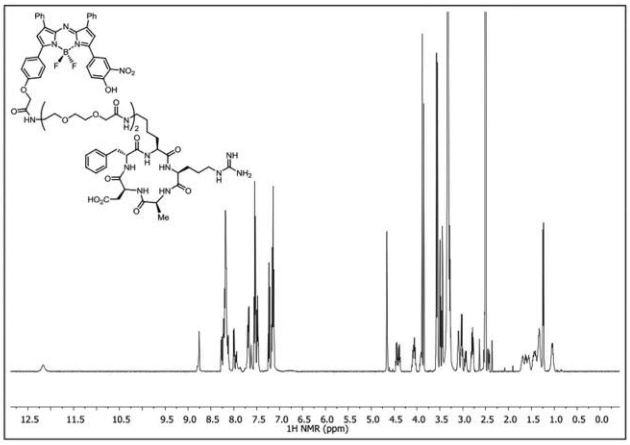

2D COSY NMR

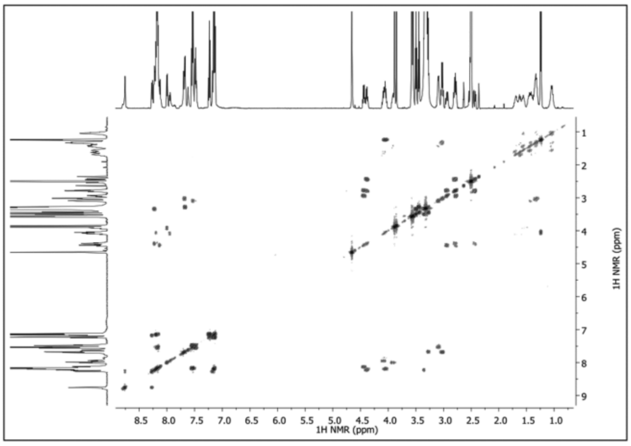

TOF Mass spectrum

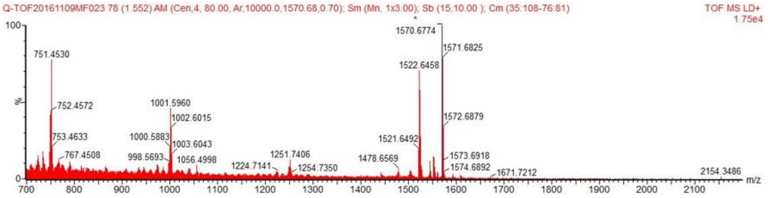

HPLC Eluent ACN:H_2O with 10 mM NH_4HCO_3; flow rate 1 mL/min; Detector wavelength 650 nm

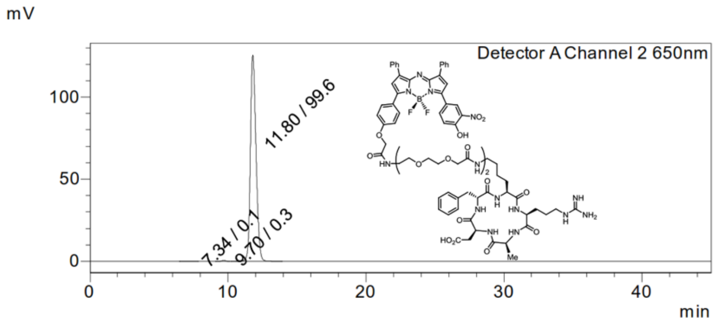

Figure S10. Analytical spectra for fluorophore **2**

Movie Legends

Movie S1. Representative confocal Z-stack of MDA-MB 231 cells acquired 2 h post incubation with **1**.

Movie S2. Representative confocal Z-stack of MDA-MB 231 cells acquired 2 h post incubation with **2**.
